# Strain and strain rate echocardiography in children with Wilson’s disease

**DOI:** 10.5830/CVJA-2016-028

**Published:** 2016

**Authors:** Karakurt Cemşit, Çelik Serkan, Selimoğlu Ayşe, Varol İlknur, Karabiber Hamza, Yoloğlu Saim

**Affiliations:** Department of Pediatric Cardiology, Faculty of Medicine, Inonu University, Malatya, Turkey; Department of Pediatric Cardiology, Faculty of Medicine, Inonu University, Malatya, Turkey; Department of Pediatric Gastroenterology, Faculty of Medicine, Inonu University, Malatya, Turkey; Department of Pediatric Gastroenterology, Faculty of Medicine, Inonu University, Malatya, Turkey; Department of Pediatric Gastroenterology, Faculty of Medicine, Inonu University, Malatya, Turkey; Department of Biostatistics, Faculty of Medicine, Inonu University, Malatya, Turkey

**Keywords:** 2D strain, strain rate echocardiography, speckle traking, Wilson's disease

## Abstract

**Objective:**

This study aimed to evaluate strain and strain rate echocardiography in children with Wilson’s disease to detect early cardiac dysfunction.

**Methods:**

In this study, 21 patients with Wilson’s disease and a control group of 20 age- and gender-matched healthy children were included. All the patients and the control group were evaluated with two-dimensional (2D) and colour-coded conventional transthoracic echocardiography by the same paediatric cardiologist using the same echocardiography machine (Vivid E9, GE Healthcare, Norway) in standard precordial positions, according to the American Society of Echocardiography recommendations. 2D strain and strain rate echocardiography were performed after the ECG probes of the echocardiography machine were adjusted for ECG monitoring. Longitudinal, transverse and radial strain, and strain rate were assessed from six basal and six mid-ventricular segments of the left ventricle, as recommended by the American Society of Echocardiography.

**Results:**

Left ventricular wall thickness, systolic and diastolic diameters, left ventricular diameters normalised to body surface area, end-systolic and end-diastolic volumes, cardiac output and cardiac index values were within normal limits and statistically similar in the patient and control groups (p < 0.05).

Global strain and strain rate: the patient group had a statistically significant lower peak A longitudinal velocity of the left basal point and peak E longitudinal velocity of the left basal (VAbasR) point, and higher global peak A longitudinal/circumferential strain rate (GSRa) compared to the corresponding values of the control group (p < 0.05).

Radial strain and strain rate: end-systolic rotation [ROT (ES)] was statistically significantly lower in the patient group (p < 0.05). Longitudinal strain and strain rate: end-systolic longitudinal strain [SLSC (ES)] and positive peak transverse strain (STSR peak P) were statistically significantly lower in the patient group (p < 0.05).

Segmental analysis showed that rotational strain measurement of the anterior and lateral segments of the patient group were statistically significantly lower than the corresponding values of the control group (p < 0.05). Segmental analysis showed statistically significantly lower values of end-systolic longitudinal strain [STSR (ES)] of the basal lateral (p < 0.05) and end-systolic longitudinal strain [SLSC (ES)] of the basal septal segment (p < 0.05) in the patient group.

End-systolic longitudinal strain [SLSC (ES)] and positive peak transverse strain (STSR peak P) were statistically significantly lower in the patient group (p < 0.05). Segmental analysis showed statistically significantly lower values of endsystolic longitudinal strain [SLSC (ES)] of the mid-anterior and basal anterior segments (p < 0.05), end-systolic longitudinal strain [STSR (ES)] measurements of the posterior and mid-posterior segments, end-systolic longitudinal displacement [DLDC (ES)] of the basal posterior, mid-posterior and mid-antero-septal segments in the patient group.

**Conclusion:**

Cardiac arrhythmias, cardiomyopathy and sudden cardiac death are rare complications but may be seen in children with Wilson’s disease due to copper accumulation in the heart tissue. Strain and strain rate echocardiography is a relatively new and useful echocardiographic technique to evaluate cardiac function and cardiac deformation abnormalities. Our study showed that despite normal systolic function, patients with Wilson’s disease showed diastolic dysfunction and regional deformation abnormalities, especially rotational strain and strain rate abnormalities.

## Objective

Wilson’s disease is an autosomal recessive metabolic liver disease related to mutation of the copper-transporting ATPase, ATP7B, an intracellular copper transporter mainly expressed in the hepatocytes.^1^,^2^ Wilson’s disease is characterised by excessive copper deposition in the body, primarily in the liver and brain, resulting from inability of the liver to excrete copper in the bile. Cardiac arrhythmias, cardiomyopathy and sudden cardiac death are rare complications but may be seen in children with Wilson’s disease due to copper accumulation in the heart tissue.^3^,^6^ The aims of our study were to determine potential differences in strain and strain rate between patients with Wilson’s disease and age-matched controls, and to detect early cardiac dysfunction.

## Methods

In this study, 21 patients with Wilson’s disease who applied to our hospital’s Paediatric Gastroenterology Department between May and October 2013 were included (α = 0.05, 1–β = 0.8, changing ratio = 0.0015). The control group consisted of 20 ageand gender-matched healthy children. Patients with any chronic disease, obesity and hypertension in addition to Wilson’s disease or history of drug use that may have affected cardiac function were excluded from the study.

Diagnosis of Wilson’s disease was made by the Paediatric Gastroenterology Department, based on the presence of signs of liver or neurological disease and the detection of Kayser– Fleischer rings, low ceruloplasmin, and elevated levels of urinary and hepatic copper. Liver biopsies were done for all patients and associated histological changes in the liver were confirmed. Before the study, approval of the ethics committee of the Medical School of Inonu University in accordance with Declaration of Helsinki was received.

Age, body weight, height and body surface area were recorded in the patient and control groups. The age at diagnosis of Wilson’s disease was also recorded in the patient group. All subjects were evaluated with ECG before echocardiographic evaluation.

All patients and controls were evaluated with two-dimensional (2D) and color-coded conventional transthoracic echocardiography by the same paediatric cardiologist, using the same echocardiography machine (Vivid E9, GE Healthcare, Norway) in the standard precordial positions, according to the American Society of Echocardiography recommendations.^7^ Left ventricular dimensions, left ventricular wall thickness, end-diastolic and end-systolic volumes, stroke volume, cardiac index, ejection fraction and fractional shortening were measured using M-mode echocardiography in the parasternal long-axis view.

2D strain and strain rate echocardiography were performed by a paediatric cardiologist after the ECG probes of the echocardiography machine (Vivid E9, GE) were adjusted for ECG monitoring. Grey images were obtained from the apical four-, three- and two-chamber, and short-axis view at the papillary muscle position using tissue harmonic imaging with frame rates of 70 per second. All the images that were obtained in the left lateral decubitus position and under ECG monitoring were stored for offline analysis.

2D strain and strain rate measurements were performed using the ECHOPAC software package. As previously described, the endocardial border was traced manually on a single end-diastolic frame and the software automatically tracked the contour on subsequent frames. Tracking accuracy was verified in real time and corrected by adjusting the region of interest or by manually correcting the contour to ensure optimal tracking. If required, region-of-interest width or smoothing functions were changed for optimal tracking. Once the contours were approved by the paediatric cardiologist, the software calculated longitudinal, transverse, radial and global strains for the respective segments. For long-axis strain and strain rate evaluation, the atrioventricular valve closure time was selected manually.

**Fig. 1:**
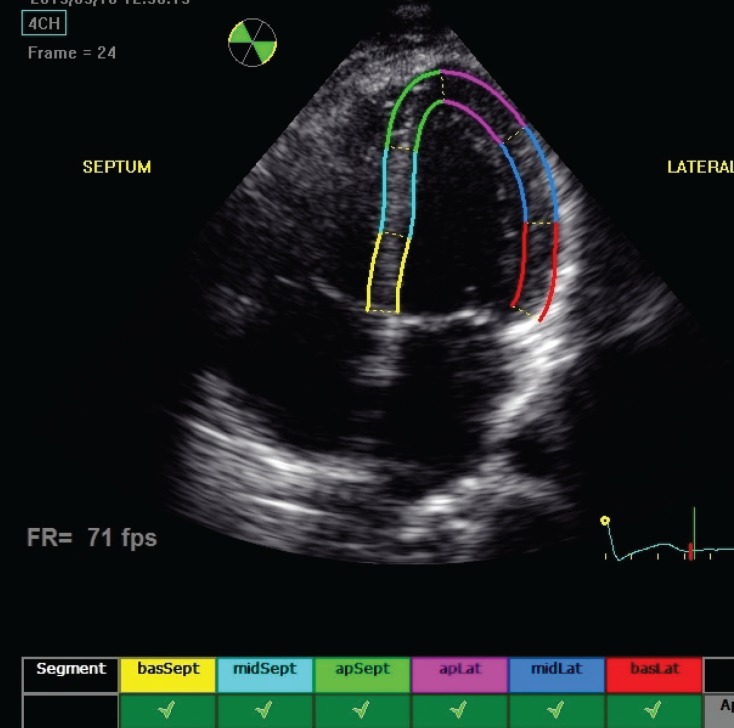
Echocardiograph shows segmental analysis of left ventricle after 2D speckle tracking from the apical fourchamber view.

**Fig. 2:**
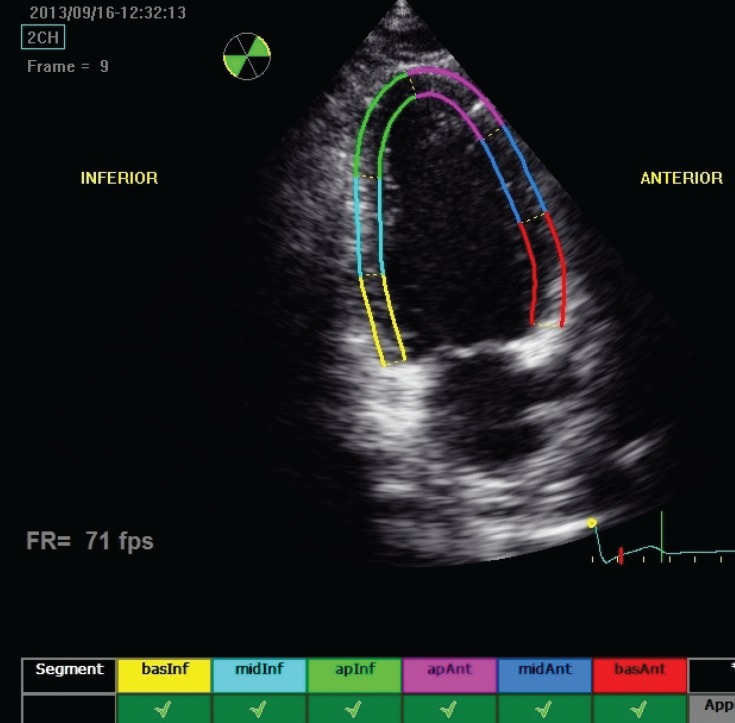
Echocardiograph shows segmental analysis of left ventricle after 2D speckle tracking from the apical two-chamber view.

**Fig. 3:**
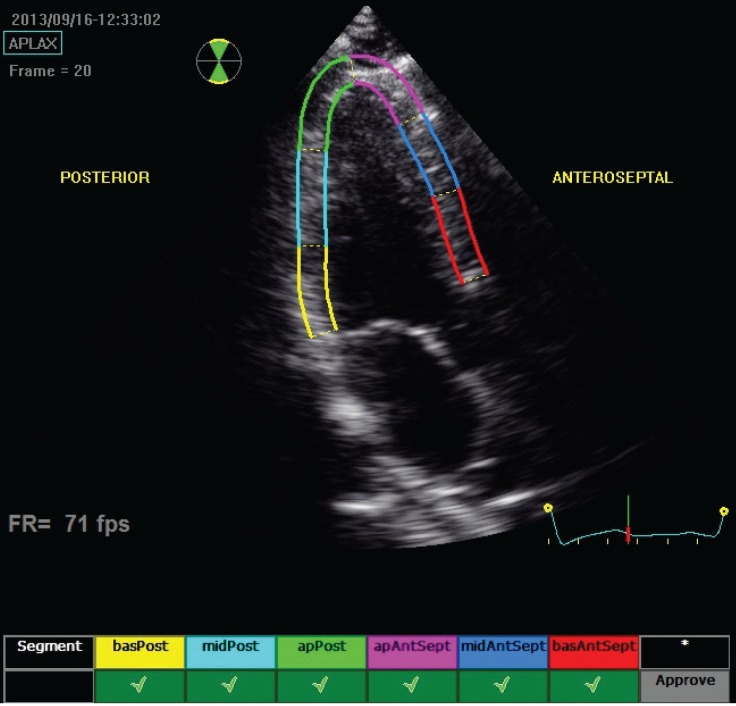
Echocardiograph shows segmental analysis of the left ventricle after 2D speckle tracking from the apical long-axis view.

**Fig. 4:**
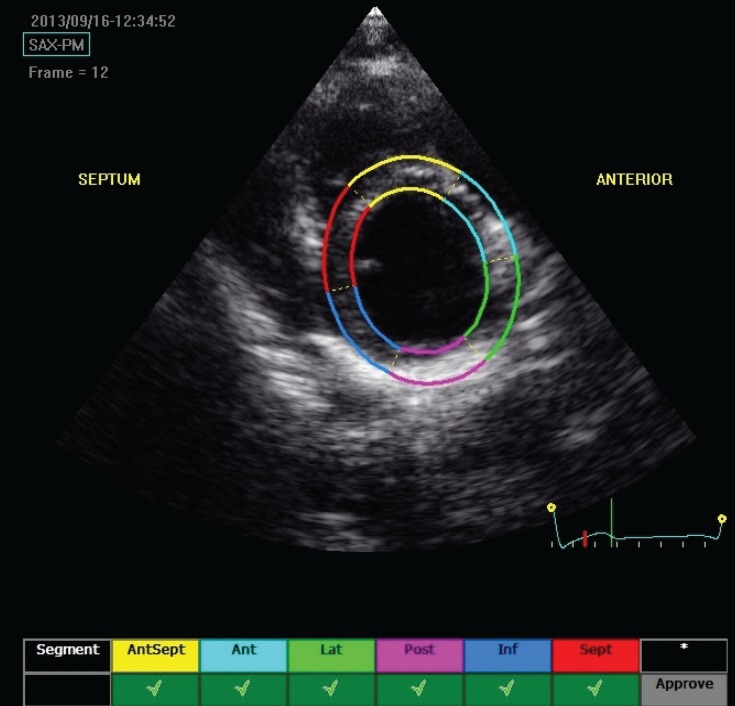
Echocardiograph shows segmental analysis of the left ventricle after 2D speckle tracking from the parasternal short-axis view.

As described, longitudinal and transverse radial strain, and strain rate were assessed from six basal and six mid-ventricular segments of the left ventricle, as recommended by the American Society of Echocardiography (Figs 1–4). This included apical, mid- and basal segments from the four-, two- and three-chamber view of the left ventricle, and anterior, septal and inferior segments from the short-axis view of the left ventricle.

## Statistical analysis

SPSS 16.0 was used for statistical analysis. The normality was tested using the Kolmogorov–Smirnov test. The unpaired t-test was used to test for differences between categorical data of the patient and control groups. The ANOVA test was used to evaluate 2D strain and strain rate values of the two groups for each segment. The LD test was applied in the second test to determine the groups that differed significantly. All the results are expressed as mean ± standard deviation; p-values < 0.05 were considered statistically significant.

## Results

The study included 21 patients with Wilson’s disease and 20 healthy age-matched children. The patient group consisted of 11 males and 10 females, and the control group, nine males and 11 females. Demographic data of the patient and control groups are shown in Table 1. There were no statistically significant differences between the patient and control groups (p > 0.05). The mean age at diagnosis was 9 ± 2.24 years (5–13) in the patient group. All the subjects had normal sinus rhythm. Wolf–Parkinson–White syndrome was detected in one patient’s ECG.

No structural heart disease was detected with conventional 2D colour-coded transthoracic echocardiography. Left ventricular wall thickness (IVSd, IVSs, LPWd, LWPDs), systolic and diastolic diameters (LVIDd, LVIDs), left ventricular diameters normalised to body surface area (LVEDd/m², LVEDs/m²), end-systolic and end-diastolic volumes (ESV, EDV), cardiac output and cardiac index values were within normal limits and statistically similar in the patient and control groups (p < 0.05). Demographic data, left ventricular wall thickness, dimensions, volumes and systolic function of the patient and control groups are shown Table 1.

**Table 1:** Demographic data of left ventricular wall thickness, dimension, volume and systolic function of Wilson’s disease patients and controls

	*Patients(n = 21)*	*controls (n = 20)*	
*Parameters*	*mean ± SD*	*mean ± SD*	*p-value*
Age (years)	11.04 ± 3 .58 (5–17)	10.53 ± 2.8 (6–16)	0.61
Gender (male/female)	11/10	9/11	0.64
Weight (kg)	38.1 ± 16.04	38.1 ± 16.04	0.84
	(14.5–68)	(21.4–67)	
Height (cm)	141.5 ± 20.2	141.1 ± 14.2	0.93
	(100–187)	(120–168)	
Body surface area (m^3^)	1.21 ± 0.33	1.22 ± 0.25	0.92
	(0.63–1.91)	(0.9–1.72)	
Age of diagnosis	9 ± 2.24	-	-
	(5–13)		
IVSd (mm)	7.14 ± 1.10	6.45 ± 1.39	0.85
IVSs (mm)	10.76 ± 1.78	9.7 ± 1.65	0.56
LPWDd (mm)	5.42 ± 1.16	5.2 ± 1.36	0.56
LPWDs (mm)	9.52 ± 2.11	8.8 ± 1.28	0.1
LVIDd (mm)	41.6 ± 6.81	42.1 ± 4.52	0.79
LVEDd/m^2^ (mm/m^2^)	35.5 ± 8.45	35.2 ± 5.16	0.92
LVEDs (mm)	24.8 ± 4.65	26.5 ± 6.09	0.32
LVEDs/m^2^ (mm/m^2^)	21.05 ± 5.08	22.19 ± 4.68	0.46
EDV (ml)	83.38 ± 28.76	79.8 ± 20.41	0.65
ESV (ml )	24.28 ± 11.89	29.5 ± 20.79	0.32
SV (ml)	60.85 ± 20.53	55.80 ± 13.65	0.36
CI (ml/min)	4.60 ± 1.32	3.98 ± 0.78	0.077
EF (%)	71.76 ± 6.51	69.90 ± 5.33	0.32
FS (%)	40.9 ± 5.62	39.1 ± 4.54	0.26

EDV: end-diastolic volume, ESV: end-systolic volume, SV: stroke volume, CI: cardiac index, EF: ejection fraction, FS: fractional shortening, CI: cardiac index.

## Global strain and strain rate

No statistically significant differences were found between the two groups for global longitudinal/circumferential strain rate, global peak systolic longitudinal/circumferential strain rate, global peak E longitudinal/circumferential strain rate, peak E longitudinal velocity of the left basal point, peak E longitudinal velocity of the right basal point, and peak A longitudinal velocity of the left basal point (cm/s).

While the control group had statistically significantly lower global peak A longitudinal/circumferential strain rates, the patient group had statistically significantly lower peak A longitudinal velocity of the left basal point and peak E longitudinal velocity of the left basal point (p < 0.05). Global strain and strain rate values of the patients and controls are shown in Table 2.

**Table 2:** Global strain and strain rate values of Wilson’s disease patients and controls

**	*Patients*	*Controls*	
**	*(n = 21)*	*(n = 20)*	
*Parameters*	*(mean ± SD)*	*(mean ± SD)*	*p-value*
GS (%)	–17.30 ± 3.22	–17.11 ± 3.69	0.70
GSRs (1/s)	–1.07 ± 0.20	–1.02 ± 0.23	0.17
GSRe (1/s)	1.54 ± 0.40	1.59 ± 0.46	0.35
GSRa (1/s)	0.74 ± 0.44	0.53 ± 0.23	0.02
VEbasL (cm/s)	–11.25 ± 2.95	–11.20 ± 2.53	0.91
VESbasR (cm/s)	–9.22 ± 3.03	–9.91 ± 3.00	0.16
VAbasL (cm/s)	–6.69 ± 2.991	–5.09 ± 1.930	0.001
VAbasR (cm/s)	–5.95 ± 2.807	–4.65 ± 2.129	0.002

GS: global peak longitudinal/circumferential strain, GSGRs: global peak systolic longitudinal/circumferential strain rate, GSRe/1s: global peak E longitudinal/ circumferential strain rate, GSRa1/s: global peak A longitudinal/circumferential strain rate, VEbasL: left basal peak E longitudinal velocity, VEbasR: right basal peak E longitudinal velocity point, VAbasL: left basal peak A longitudinal velocity, VAbasR: right basal peak A longitudinal velocity.

## Radial strain and strain rate

No statistically significant differences were found between the two groups for the most negative peak circumferential strain (SLSC peak G), negative systolic peak circumferential strain (SLSC peak S), positive systolic peak circumferential strain (SLSC peak P), end-systolic circumferential strain [SLSC (ES)] and end-systolic radial displacement [DTDR (ES)] (p < 0.05).

End-systolic rotation [ROT (ES)] was statistically significantly lower in the patient group (p < 0.05). Segmental analysis showed that rotational strain measurement of the anterior segment was statistically significantly lower (–0.78 ± 3.16) in the patient group (p = 0.019). The lateral segment was also statistically significantly lower in the patient group (–1.17 ± 3.17) (p = 0.14). Radial strain and strain rate measurements of patients and controls are shown in Table 3. Radial strain and strain rate values according to the segments are shown in Table 4.

**Table 3:** Radial strain and strain rate measurements of Wilson’s disease patients and controls

**	*Patients (n = 21)*	Controls (n = 20)**	
*Strian*	*(mean ± SD)*	*(mean ± SD)*	*p-value*
SLSC peak G (%)	–16.45 ± 10.74	–16.09 ± 9.85	0.77
SLSC peak S (%)	–15.22 ± 11.57	–14.64 ± 11.38	0.67
SLSC peak P (%)	2.60 ± 5.09	2.75 ± 5.46	0.82
SLSC (ES) (%)	–14.20 ± 12.41	–13.71 ± 12.89	0.74
STSR (ES) (%)	41.04 ± 18.29	44.92 ± 17.47	0.72
DTDR (ES) (mm)	5.145 ± 2.54	5.01 ± 2.48	0.65
ROT (ES) (°)	–4.49 ± 4.53	–2.84 ± 5.06	0.004

SLSC peak G: the most negative peak circumferential strain, SLSC peak S: negative systolic peak circumferential strain, SLSC peak P: positive systolic peak circumferential strain, SLSC (ES): end-systolic circumferential strain, DTDR (ES): end-systolic radial displacement, ROT (ES): end-systolic rotation.

**Table 4:** Radial strain and strain rate values according to segment

*Strain according to segment*	*Patients (n = 21)*	*Controls (n = 20*)	
	(mean ± SD)	(mean ± SD)	p-value
*SLSC peak S (%)*			
Anterior	–9.55 ± 7.79	–9.71 ± 8.90	0.94
Lateral	–3.68 ± 5.75	–4.34 ± 5.76	0.69
Posterior	–6.60 ± 8.31	–3.48 ± 8.57	0.21
Inferior	–19.95 ± 6.84	–20.01 ± 5.32	0.97
Septal	–27.83 ± 6.57	–27.03 ± 4.63	0.64
Anterior septal	–23.85 ± 7.69	–23.28 ± 6.11	0.78
*SLSC peak P (%)*			
Anterior	2.33 ± 2.89	2.18 ± 2.34	0.84
Lateral	4.31 ± 4.33	5.56 ± 6.30	0.41
Posterior	4.44 ± 6.25	7.37 ± 9.59	0.20
Inferior	1.71 ± 4.20	0.69 ± 0.90	0.29
Septal	1.31 ± 6.10	0.25 ± 0.56	0.44
Anterior	1.48 ± 5.37	0.43 ± 0.984	0.39
*SLSC (ES) (%)*			
Anterior	–9.21 ± 7.78	–9.50 ± 8.93	0.90
Lateral	–1.93 ± 7.40	–2.07 ± 8.98	0.95
Posterior	–4.49 ± 10.16	–0.89 ± 12.05	0.26
Inferior	–19.28 ± 7.42	–19.77 ± 5.35	0.80
Septal	–27.05 ± 7.05	–26.87 ± 4.62	0.91
Anterior septal	–23.46 ± 7.80	–23.14 ± 6.03	0.88
*STSR (ES)*			
Anterior	42.87 ± 19.56	44.36 ± 16.27	0.78
Lateral	39.94 ± 17.33	41.01 ± 16.22	0.83
Posterior	36.04 ± 15.37	39.35 ± 19.90	0.52
Inferior	38.72 ± 17.77	48.70 ± 19.12	0.06
Septal	43.58 ± 19.31	48.87 ± 16.59	0.32
Anterior septal	45.29 ± 20.14	47.21 ± 16.18	0.72
*DTDR (ES) (mm)*			
Anterior	6.70 ± 1.93	6.14 ± 2.10	0.34
Lateral	7.75 ± 1.74	6.98 ± 2.25	0.18
Posterior	6.16 ± 1.55	5.79 ± 2.04	0.48
Inferior	3.34 ± 2.02	3.88 ± 2.23	0.39
Septal	2.70 ± 1.64	3.146 ± 2.16	0.41
Anterior septal	4.22 ± 1.58	4.10 ± 1.78	0.81
*ROT (ES ) (°)*			
Anterior	–0.78 ± 3.16	1.33 ± 2.72	0.01
Lateral	–3.60 ± 3.30	–1.17 ± 3.17	0.01
Posterior	–7.04 ± 3.89	–5.48 ± 4.79	0.22
Inferior	–8.76 ± 4.34	–7.75 ± 5.36	0.47
Septal	–5.07 ± 3.49	–4.13 ± 4.01	0.38
Anterior septal	–1.45 ± 3.06	0.13 ± 3.181	0.09

SLSC peak S: negative systolic peak circumferential strain, STSR (ES): end-systolic longitudinal strain, SLSC peak P: positive systolic peak circumferential strain, SLSC (ES): end-systolic circumferential strain, DTDR (ES): end-systolic radial displacement, ROT (ES): end-systolic rotation (°).

## Longitudinal and transverse strain and strain rate

Four chambers: longitudinal and transverse strain and strain rate measurements are shown in Table 5. No statistically significant differences were found between the two groups for the most negative peak longitudinal strain (SLSC peak G), negative systolic peak longitudinal strain (SLSC peak S), positive systolic peak longitudinal strain (SLSC peak P), the most negative peak transverse strain (STSR peak G), end-systolic longitudinal strain [STSR (ES)] and end-systolic longitudinal displacement [DLDC (ES)].

**Table 5:** Longitudinal and transverse strain and strain rate values according to segment from the four-chamber view

*Strain according to segment*	*Patients (n = 21)*	**Controls (n = 20)	**
**	*(mean ± SD)*	*(mean ± SD)*	*p-value*
SLSC peak G (%)			
Basal septal	–21.07 ± 2.32	–21.46 ± 2.62	0.57
Mid-septal	–20.52 ± 2.20	–20.67 ± 2.17	0.80
Apical septal	–17.56 ± 3.72	–18.20 ± 3.70	0.54
Apical lateral	–15.56 ± 4.59	–16.91 ± 3.75	0.26
Mid-lateral	–14.42 ± 4.35	–15.89 ± 4.24	0.23
Basal lateral	–14.10 ± 5.66	–14.07 ± 5.44	0.98
SLSC peak S (%)			
Basal septal	–20.74 ± 2.50	–21.26 ± 2.67	0.47
Mid-septal	–20.40 ± 2.27	–20.56 ± 2.22	0.79
Apical septal	–17.04 ± 3.72	–17.57 ± 3.93	0.62
Apical lateral	–15.30 ± 4.60	–16.67 ± 3.93	0.26
Mid-lateral	–13.96 ± 4.70	–15.58 ± 4.26	0.20
Basal lateral	–12.79 ± 6.79	–12.42 ± 7.98	0.86
SLSC peak P (%)			
Basal septal	0.29 ± 0.47	0.26 ± 0.41	0.83
Mid-septal	0.07 ± 0.29	0.03 ± 0.09	0.48
Apical septal	0.25 ± 1.29	0.08 ± 0.31	0.53
Apical lateral	0.23 ± 1.20	0.001 ± 0.03	0.35
Mid-lateral	0.38 ± 0.72	0.22 ± 0.55	0.38
Basal lateral	2.76 ± 5.00	3.11 ± 4.03	0.78
STSR peak P (%)			
Basal septal	32.31 ± 18.40	37.90 ± 20.18	0.30
Mid-septal	26.00 ± 15.45	31.24 ± 14.79	0.22
Apical septal	21.90 ± 14.32	26.700 ± 11.13	0.19
Apical lateral	19.82 ± 14.28	25.82 ± 14.04	0.14
Mid-lateral	20.38 ± 14.76	27.33 ± 17.61	0.13
Basal lateral	22.64 ± 17.76	31.21 ± 21.17	0.012
STSR peak G (%)			
Basal septal	–20.53 ± 2.50	–21.12 ± 2.68	0.42
Mid-septal	–20.32 ± 2.26	–20.51 ± 2.17	0.76
Apical septal	–16.95 ± 3.81	–17.49 ± 3.86	0.61
Apical lateral	–15.14 ± 4.72	–16.53 ± 3.90	0.26
Mid-lateral	–13.66 ± 5.11	–15.44 ± 4.29	0.18
Basal lateral	–11.58 ± 9.45	–12.64 ± 6.89	0.17
SLSC (ES) (%)			
Basal septal	28.43 ± 20.12	35.12 ± 21.41	0.25
Mid-septal	23.91 ± 16.32	30.12 ± 15.08	0.16
Apical septal	20.64 ± 14.76	26.02 ± 11.08	0.15
Apical lateral	18.58 ± 14.74	24.56 ± 14.35	0.15
Mid-lateral	17.53 ± 16.27	24.30 ± 18.47	0.17
Basal lateral	16.90 ± 21.08	24.79 ± 22.54	0.69
STSR (ES) (%)			
Basal septal	12.14 ± 2.18	12.18 ± 2.69	0.95
Mid-septa	6.64 ± 1.52	6.73 ± 2.23	0.87
Apical septal	1.60 ± 0.74	1.81 ± 1.25	0.45
Apical lateral	2.88 ± 1.32	2.78 ± 0.96	0.77
			
Basal lateral	11.06 ± 3.50	11.33 ± 2.27	0.66
DLDC (ES) (mm)			
			
Basal septal	1.85 ± 1.74	3.16 ± 1.89	0.014
Mid-septal	1.97 ± 1.34	2.81 ± 1.85	0.06
Apical septal	1.55 ± 1.06	2.01 ± 1.15	0.14
Apical lateral	1.89 ± 0.91	2.05 ± 0.59	0.46
Mid-lateral	3.66 ± 1.26	3.52 ± 1.073	0.66
Basal lateral	5.97 ± 1.77	5.51 ± 1.64	0.35

SLSC peak G: the most negative peak longitudinal strain, SLSC peak S: negative systolic peak longitudinal strain, SLSC peak P: positive systolic peak longitudinal strain, SLSC (ES): end-systolic longitudinal strain, STSR peak P: positive peak transverse strain, STSR peak G: the most negative peak transverse strain, STSR (ES): end-systolic longitudinal strain, DLDC (ES): end-systolic longitudinal displacement.

End-systolic longitudinal strain [SLSC (ES)] and positive peak transverse strain (STSR peak P) were statistically significantly lower in the patient group (p < 0.05). Segmental analysis showed statistically significantly lower levels of end-systolic longitudinal strain [STSR (ES)] of the basal lateral (p < 0.05), and statistically significantly lower levels of end-systolic longitudinal strain [SLSC (ES)] of the basal septal segment (p < 0.05) in the patient group. Longitudinal and transverse strain and strain rate measurements from four and two chambers and the apical longaxis views are shown in Table 6.

**Table 6:** Longitudinal and transverse strain and strain rate values according to segment from the four-chamber view

*Strain according to segment*	*Patients (n = 21)*	*Controls (n = 20)*	**
**	*(mean ± SD)*	*(mean ± SD)*	*p-value*
Four-chamber view			
SLSC peak G (%)	–17.21 ± 4.82	–17.87 ± 4.55	0.22
SLSC peak S (%)	–16.70 ± 5.27	–17.34 ± 5.39	0.29
SLSC peak P (%)	0.66 ± 2.36	0.62 ± 1.99	0.84
STSR peak P (%)	23.84 ± 16.25	30.03 ± 17.05	0.001
STSR peak G (%)	–16.36 ± 6.11	–17.29 ± 5.08	0.15
SLSC (ES) (%)	21.00 ± 17.61	27.49 ± 17.75	0.002
STSR (ES) (%)	6.93 ± 4.41	7.05 ± 4.32	0.80
DLDC (ES) (mm)	2.819 ± 2.08	3.180 ± 1.84	0.11
Two-chamber view		–	
SLSC peak G (%)	18.73 ± 5.44	–17.56 ± 7.24	0.13
SLSC peak S (%)	–18.46 ± 5.48	–17.34 ± 7.54	0.16
SLSC peak P (%)	0.38 ± 0.87	0.72 ± 2.03	0.07
STSR peak P (%)	25.55 ± 18.21	31.68 ± 17.63	0.005
STSR peak G (%)	–18.32 ± 5.42	–18.32 ± 5.42	0.16
SLSC (ES) (%)	20.46 ± 19.14	29.17 ± 18.67	0.001
STSR (ES) (%)	7.25 ± 5.11	6.72 ± 5.25	0.40
DLDC (ES) (mm)	3.44 ± 2.25	3.35 ± 2.23	0.73
Apical long-axis view (APLAX)			
SLSC peak G (%)	–17.73 ± 5.92	–17.18 ± 5.36	0.36
SLSC peak S (%)	–17.38 ± 5.84	–16.97 ± 5.43	0.49
SLSC peak P (%)	0.62 ± 3.76	0.54 ± 1.18	0.80
STSR peak P (%)	24.15 ± 1.92	26.40 ± 20.12	0.55
STSR peak G (%)	–17.21 ± 6.08	–16.84 ± 5.45	0.55
SLSC (ES) (%)	20.02 ± 22.72	23.04 ± 21.12	0.20
STSR (ES) (%)	7.35 ± 5.11	6.85 ± 4.52	0.33
DLDC (ES) (mm)	2.71 ± 2.10	2.68 ± 1.69	0.89

SLSC peak G: the most negative peak longitudinal strain, SLSC peak S: negative systolic peak longitudinal strain, SLSC peak P: positive systolic peak longitudinal strain, SLSC (ES): end-systolic longitudinal strain, STSR peak P: positive peak transverse strain, STSR peak G: the most negative peak transvers strain, STSR (ES): end-systolic longitudinal strain, DLDC (ES): end-systolic longitudinal displacement (mm).

Two chambers: there were no statistically significant differences found between the two groups for the most negative peak longitudinal strain (SLSC peak G), negative systolic peak longitudinal strain (SLSC peak S), positive systolic peak longitudinal strain (SLSC peak P), the most negative peak transverse strain (STSR peak G), end-systolic longitudinal strain [STSR (ES)] and end-systolic longitudinal displacement [DLDC (ES)].

End-systolic longitudinal strain [SLSC (ES)] and positive peak transverse strain (STSR peak P) were statistically significantly lower in the patient group (p < 0.05). Segmental analysis showed statistically significantly lower values of end-systolic longitudinal strain [SLSC (ES)] of the mid-anterior and basal anterior segments (p < 0.05) in the patient group. The most negative peak longitudinal strain (SLSC peak S) value was –18.00 ± 3.33 in the mid-anterior segment of the patient group and –14.9723 ± 6.23886 in the mid-anterior segment of the control group (p < 0.05) (Tables 7,8).

**Table 7:** Longitudinal and transverse strain and strain rate values according to segment from the apical long-axis view

*Strain according to segment*	*Patients (n = 21)*	**Controls (n = 20)	**
	**(mean ± SD)	**(mean ± SD)	*p-value*
SLSC peak G (%)			
Basal inferior	–21.80 ± 4.37	–21.14 ± 6.86	0.70
Mid-inferior	–21.24 ± 3.56	–20.69 ± 6.95	0.73
Apical inferior	–17.40 ± 3.73	–17.65 ± 6.61	0.87
Apical anterior	–11.41 ± 4.04	–12.17 ± 5.90	0.62
Mid-anterior	–18.00 ± 3.33	–14.97 ± 6.23	0.047
Basal anterior	–22.22 ± 5.04	–18.75 ± 7.16	0.06
SLSC peak S (%)			
Basal inferior	–21.75 ± 0.35	–21.20 ± 6.26	0.73
Mid-inferior	–21.18 ± 3.61	–20.62 ± 6.99	0.73
Apical inferior	–16.97 ± 3.72	–17.34 ± 7.44	0.83
Apical anterior	–10.99 ± 3.97	–11.75 ± 6.44	0.63
Mid-anterior	–17.72 ± 3.30	–17.72 ± 3.30	0.06
Basal anterior	–21.82 ± 4.94	–18.35 ± 7.63	0.07
SLSC peak P (%)			
Basal inferior	0.33 ± 0.49	0.62 ± 1.034	0.23
Mid-inferior	0.06 ± 0.18	0.52 ± 1.98	0.27
Apical inferior	0.46 ± 0.66	1.03 ± 3.14	0.39
Apical anterior	0.65 ±1.11	1.01 ± 2.53	0.55
Mid-anterior	0.20 ± 0.52	0.55 ± 1.67	0.35
Basal anterior	0.61 ± 1.50	0.63 ± 1.18	0.95
STSR peak P (%)			
Basal inferior	30.87 ± 12.70	30.60 ± 11.08	0.93
Mid-inferior	24.16 ± 8.65	26.84 ± 9.22	26.84 ± 9.22
Apical inferior	20.89 ± 7.31	26.22 ± 11.44	0.06
Apical anterior	21.55 ± 13.51	29.40 ± 16.40	0.09
Mid-anterior	25.47 ± 23.34	35.20 ± 21.54	0.15
Basal anterior	30.20 ± 30.81	41.83 ± 26.16	0.180
STSR peak G (%)			
Basal inferior	–21.55 ± 4.19	–20.82 ± 6.76	0.66
Mid-inferior	–21.09 ± 3.52	–20.40 ± 7.23	0.68
Apical inferior	–16.92 ± 3.72	–17.32 ± 7.19	0.81
Apical anterior	–10.92 ± 3.95	–11.73 ± 6.37	0.61
Mid-anterior	–17.53 ± 3.26	–14.735 ± 6.58	0.07
Basal anterior	–21.61 ± 5.00	–18.36 ± 7.37	0.09
SLSC (ES) (%)			
Basal inferior	24.62 ± 16.37	25.58 ± 13.77	0.83
Mid-inferior	21.18 ± 10.41	24.35 ± 11.35	0.33
Apical inferior	19.02 ± 8.22	24.90 ± 12.82	0.07
Apical anterior	18.972 ± 13.33	28.12 ± 17.37	0.05
Mid-anterior	19.41 ± 23.34	33.33 ± 22.37	0.047
Basal anterior	19.51 ± 32.88	38.74 ± 26.77	0.038
STSR (ES) (%)			
Basal inferior	13.93 ± 3.22	13.21 ± 4.30	0.53
Mid-inferior	7.70 ± 1.99	7.55 ± 2.58	0.82
Apical inferior	2.06 ± 0.97	2.14 ± 1.27	0.811
Apical anterior	1.67 ± 1.15	1.67 ± 1.83	0.999
Mid-anterior	5.69 ± 1.81	5.24 ± 3.03	0.55
Basal anterior	12.19 ± 2.72	10.50 ± 4.68	0.14
DLDC (ES) (mm)			
Basal inferior	4.40 ± 1.88	4.46 ± 2.09	0.91
Mid-inferior	4.07 ± 1.71	3.89 ± 1.83	0.72
Apical inferior	2.49 ± 1.42	2.54 ± 1.30	0.90
Apical anterior	1.42 ± 0.94	1.64 ± 1.04	0.45
Mid-anterior	2.58 ± 1.57	2.5523 ± 1.75	0.94
Basal anterior	5.59 ± 2.74	5.00 ± 2.96	0.48

SLSC peak G: the most negative peak longitudinal strain, SLSC peak S: negative systolic peak longitudinal strain, SLSC peak P: positive systolic peak longitudinal strain, SLSC (ES): end-systolic longitudinal strain, STSR peak P: positive peak transverse strain, STSR peak G: the most negative peak transverse strain, STSR (ES): end-systolic longitudinal strain, DLDC (ES): end-systolic longitudinal displacement.

**Table 8:** Longitudinal and transverse strain and strain rate values according to segment from the apical long-axis view

*Strain according to segment*	*Patients (n = 21)*	*Controls (n = 20)*	**
**	*(mean ± SD)*	*(mean ± SD)*	*p-value*
SLSCC peak G (%)			
Basal anterior septal	–19.99 ± 2.75	–18.63 ± 5.80	0.24
0.24	–16.60 ± 11.17	–15.34 ± 5.24	0.58
Mid-posterior	–18.27 ± 3.83	–17.08 ± 3.20	0.20
Apical posterior	–17.94 ± 4.47	–17.73 ± 4.48	0.86
Apical anterior septal	–15.33 ± 5.55	–16.56 ± 6.86	0.46
Mid-anterior septal	–18.01 ± 2.58	–17.72 ± 5.69	0.80
SLSC peak S (%)			
Basal anterior septal	–19.68 ± 3.06	–18.45 ± 5.81	0.31
Basal posterior	–16.29 ± 10.74	–15.06 ± 5.42	0.59
Mid-posterior	–17.98 ± 4.10	–17.018 ± 3.14	0.32
Apical posterior	–17.39 ± 4.56	–17.44 ± 4.80	0.96
Apical anterior septal	–14.91 ± 5.32	–16.21 ± 6.88	0.43
Mid-anterior septal	–17.82 ± 2.71	–17.64 ± 5.65	0.87
SLSC peak P (%)			
Basal anterior septal	0.25 ± 0.81	0.125 ± 0.28	0.42
Basal posterior	2.88 ± 8.90	2.07 ± 2.08	0.64
Mid-posterior	0.16 ± 0.49	0.43 ± 0.69	0.08
Apical posterior	0.15 ± 0.29	0.17 ± 0.42	0.84
Apical anterior septal	0.22 ± 0.36	0.30 ± 0.68	0.61
Mid-anterior septal	0.02 ± 0.06	0.14 ± 0.35	0.07
STSR peak P (%)			
Basal anterior septal	21.30 ± 22.17	29.87 ± 24.35	0.16
Basal posterior	35.91 ± 26.93	34.86 ± 25.61	0.88
Mid-posterior	27.98 ± 23.49	27.18 ± 18.86	0.88
Apical posterior	21.76 ± 18.45	21.56 ± 14.17	0.96
Apical anterior septal	19.14 ± 16.83	20.87 ± 13.64	0.67
Mid-anterior septal	18.56 ± 18.17	24.07 ± 19.07	0.26
STSR peak G (%)			
Basal anterior septal	–19.54 ± 3.04	–18.38 ± 5.85	0.39
Basal posterior	–16.04 ± 11.32	–14.81 ± 5.47	0.60
Mid-posterior	–17.89 ± 4.14	–16.87 ± 3.17	0.29
Apical posterior	–17.19 ± 4.63	–17.30 ± 4.75	0.92
Apical anterior septal	–14.66 ± 5.51	–16.09 ± 6.82	0.39
Mid-anterior septal	–17.68 ± 2.84	–17.59 ± 5.66	0.93
SLSC (ES) (%)			
Basal anterior septal	14.59 ± 24.85	24.53 ± 25.34	0.13
Basal posterior	28.05 ± 27.70	28.55 ± 28.82	0.94
Mid-posterior	24.35 ± 23.59	23.42 ± 21.06	0.87
Apical posterior	20.06 ± 19.09	20.12 ± 14.95	0.99
Apical anterior septal	17.58 ± 17.78	19.77 ± 14.08	0.61
Mid-anterior septal	15.53 ± 20.06	21.84 ± 19.12	0.22
STSR (ES) (%)			
Basal anterior septal	10.95 ± 3.58	11.50 ± 3.49	0.55
Basal posterior	13.75 ± 3.59	11.37 ± 2.26	0.004
Mid-posterior	8.81 ± 2.56	7.20 ± 1.59	0.0006
Apical posterior	3.23 ± 1.26	2.27 ± 1.216	0.005
Apical anterior septal	1.28 ± 1.61	2.21 ± 2.46	0.10
Mid-anterior septal	5.55 ± 2.96	6.56 ± 3.34	0.23
DLDC (ES) (mm)			
Basal anterior septal	1.77 ± 2.00	2.62 ± 2.11	0.11
Basal posterior	4.91 ± 2.08	3.57 ± 1.57	0.008
Mid-posterior	3.97 ± 1.59	2.95 ± 1.37	0.012
Apical posterior	2.46 ± 1.34	2.24 ± 1.31	0.53
Apical anterior septal	1.56 ± 1.37	2.17 ± 1.55	0.12
Mid-anterior septal	1.55 ± 1.50	2.54 ± 1.81	0.027

SLSC peak G: the most negative peak longitudinal strain, SLSC peak S: negative systolic peak longitudinal strain, SLSC peak P: positive systolic peak longitudinal strain, SLSC (ES): end-systolic longitudinal strain, STSR peak P: positive peak transverse strain, STSR peak G: the most negative peak transverse strain, STSR (ES): end-systolic longitudinal strain, DLDC (ES): end-systolic longitudinal displacement.

Apical long-axis (APLAX): there were no statistically significant differences found between the two groups for the most negative peak longitudinal strain (SLSC peak G), negative systolic peak longitudinal strain (SLSC peak S), positive systolic peak longitudinal strain (SLSC peak P), the most negative peak transverse strain (STSR peak G), end-systolic longitudinal strain [STSR (ES)], end-systolic longitudinal displacement [DLDC(ES)] end-systolic longitudinal strain [SLSC (ES)] and positive peak transverse strain (STSR peak P).

Segmental analysis showed that end-systolic longitudinal strain [STSR (ES)] measurements of the basal posterior and mid-posterior segments were statistically significantly lower in the patient group (p < 0.05). End-systolic longitudinal displacement [DLDC (ES)] of the basal posterior, mid-posterior and mid-anterior septal segments were statistically significantly lower in the patient group (p < 0.05).

## Discussion

Wilson’s disease is an autosomal recessive inherited disease, characterised by excessive copper storage in the body, especially the liver and brain, and also in the heart tissue due to reduced biliary copper excretion secondary to loss of the mutation for copper-transporting ATPase (ATP7B). Wilson’s disease, characterised by excessive copper deposition in the body, primarily in the liver and brain, results from an inability of the liver to excrete copper in the bile.^1^,^2^

Cardiac involvement of Wilson’s disease has not been sufficiently investigated. Electrocardiographic abnormalities, cardiac arrhythmias, cardiomyopathy, autonomic dysfunction and sudden cardiac death are rare complications but may be seen in children with Wilson’s disease due to copper accumulation in the heart tissue.^3^,^5^

Arat et al. showed increased P-wave dispersion in adults with cardiologically asymptomatic Wilson’s disease.^6^ In another study on adults, electrocardiographic abnormalities, including left ventricular hypertrophy, early repolarisation, biventricular hypertrophy, premature atrial or ventricular contractions, atrial fibrillation, sino-atrial block and Mobitz type 1 atrioventricular block were detected in 34% of the patients.^6^ Electrocardiographic abnormalities are not uncommon in Wilson’s disease and are presumably related to an underlying cardiomyopathy due to deposition of copper in the heart.^5^

We previously evaluated 22 children with Wilson’s disease with 24-hour ECG monitoring and our study showed that one patient had first-degree atrioventricular block, one had frequent sinus exit block, and four had rare ventricular ectopic beat.^8^ In our study, all the patients were assessed with ECG before echocardiographic evaluation. Only one patient had Wolf–Parkinson–White pattern, but there were no arrhythmias or ECG abnormalities in any of the patients. We assumed that ECG abnormalities develop over a long period or in untreated patients.

 The major pathological findings of the myocardium in Wilson’s disease included the presence of interstitial and myocardial fibrosis, focal myocarditis and cardiac hypertrophy, conduction tissue degeneration, and early atherosclerosis due to the toxic effect of copper, leading to mitochondrial injury and lipid peroxidation, which is caused by reactive free oxygen radicals. Hlubocka et al. suggested that cardiac changes seen in Wilson’s disease are not only related to copper accumulation but also to free oxygen radicals.^9^ Therefore echocardiography is an important tool to assess asymptomatic Wilson’s patients. In this study, left ventricular wall thickness was increased and left ventricular end-diastolic diameter was decreased in patients with Wilson’s disease and the differences were statistically comparable to the results of the control group. Measurement of local and global myocardial function using non-invasive methods is a major aim in clinical cardiology.^10^ Cardiac systolic function may deteriorate in prolonged disease or in untreated patients.

In our study, systolic function of the left ventricle, wall thickness, left ventricular diameters, volumes, and cardiac index measurements were within normal limits. There were no statistically significant differences between the Wilson’s disease patients and the control group for left ventricular systolic function, wall thickness, diameter, volume and cardiac index (p < 0.05). Our patients were relatively young and the mean age at diagnosis was 9 ± 2.24 years, so their time from diagnosis was relatively short. Cardiac systolic function may deteriorate in the long term or due to serious disease.

Tissue Doppler echocardiography, strain and strain rate echocardiography are relatively novel echocardiographic techniques and important tools to assess asymptomatic patients.^11^,^14^ Tissue Doppler imaging, which has recently allowed a detailed examination of cardiac function, is widely used to evaluate children with various conditions.^14^,^17^ Our previous study showed early diastolic dysfunction in patients with Wilson’s disease using tissue Doppler echocardiography. Despite its reliability, tissue Doppler echocardiography cannot show regional deformation and regional deformation abnormalities.

Difficulties with angle dependency of tissue Doppler imaging, the effects of preload, and the translational motion of the heart were overcome by strain and strain rate echocardiography, which were then adopted as new models in the assessment of myocardial performance and local deformation properties.^13^,^15^ Strain imaging, based on speckle tracking, in particular, enabled assessment of myocardial motion and deformation irrespective of angle and geometry, allowing an improved examination of the myocardial mechanics.

Our hospital is a liver transplantation centre, therefore, we aimed to assess a new group of children with Wilson’s disease using 2D strain and strain rate echocardiography. Strain and strain rate echocardiography are superior to tissue Doppler echocardiography in the evaluation of regional myocardial function because they are not affected by the translation and stretching of neighbouring myocardial segments.^16^ 2D strain and strain rate echocardiography also can assess different clinical conditions, such as hypertension, obesity, post exercise, Marfan syndrome, healthy children and infants.^15^,^18^

To our knowledge, the literature presents no studies showing early detection of subclinical cardiac dysfunction in children with Wilson’s disease using 2D strain and strain rate echocardiography. Our study showed that among the global strain and strain rate parameters, Wilson’s disease patients had lower peak A longitudinal velocity of the left basal point (VAbasL) and peak E longitudinal velocity of the left basal (VEbasR) point than those of the control group (p < 0.05). The patients also had statistically significantly higher global peak A longitudinal/circumferential strain rate (GSRa) (p < 0.05).

Longitudinal strain and strain rate from the four-chamber view showed that end-systolic longitudinal strain [SLSC (ES)] and positive peak transverse strain (STSR peak P) were statistically significantly lower in the patient group (p < 0.05). Radial strain and strain rate analysis showed that end-systolic rotation [ROT (ES)] was statistically significantly lower in the patient group (p < 0.05). Longitudinal strain and strain rate from the two-chamber view showed that end-systolic longitudinal strain [SLSC (ES)] and positive peak transverse strain (STSR peak P) were statistically significantly lower in the patient group (p < 0.05).

Segmental analysis also showed that rotational strain measurement of the anterior segment of the patient group, end-systolic longitudinal strain [STSR (ES)] of the basal lateral (p < 0.05), mid-anterior and basal anterior segments, and end-systolic longitudinal strain [SLSC (ES)] of the basal septal and lateral segments, and the most negative peak longitudinal strain (SLSC peak S) of the mid-anterior segment were statistically significant lower in the patient group (p < 0.05). Our study showed that in the early stages of Wilson’s disease, diastolic dysfunction due to copper accumulation may be heterogenous but in the long term, the heart could be affected globally. Early diastolic dysfunction can be detected using strain and strain rate echocardiography.

## Conclusion

Cardiac arrhythmias, cardiomyopathy and sudden cardiac death are rare complications but may be seen in children with Wilson’s disease due to copper accumulation in the heart tissue. Strain and strain rate echocardiography is a relatively new and useful echocardiographic technique to evaluate cardiac function and cardiac deformation abnormalities. In our study, despite normal systolic function, the patients with Wilson’s disease showed diastolic dysfunction and regional deformation abnormalities, especially rotational strain and strain rate abnormalities. We suggest that diastolic dysfunction, and rotational strain and strain rate abnormalities in Wilson’s disease may be progressive in untreated patients.
